# Flexural Damage Diagnosis in Reinforced Concrete Beams Using a Wireless Admittance Monitoring System—Tests and Finite Element Analysis

**DOI:** 10.3390/s21030679

**Published:** 2021-01-20

**Authors:** Constantin E. Chalioris, Violetta K. Kytinou, Maristella E. Voutetaki, Chris G. Karayannis

**Affiliations:** 1 Laboratory of Reinforced Concrete and Seismic Design of Structures, Civil Engineering Department, School of Engineering, Democritus University of Thrace, 67100 Xanthi, Greece; vkytinou@civil.duth.gr (V.K.K.); karayan@civil.duth.gr (C.G.K.); 2 Structural Science and Technology Division, Architectural Engineering Department, School of Engineering, Democritus University of Thrace, 67100 Xanthi, Greece; mvouteta@arch.duth.gr

**Keywords:** piezoelectric of lead zirconate titanate (PZT), structural health monitoring (SHM), reinforced concrete (RC), tests, damage diagnosis

## Abstract

The utilization and effectiveness of a custom-made, portable and low-cost structural health monitoring (SHM) system that implements the PZT-based electro-mechanical admittance (EMA) methodology for the detection and evaluation of the damage of flexural reinforced concrete (RC) beams is presented. Tests of large-scale beams under monotonic and cyclic reversal-imposed deformations have been carried out using an integrated wireless impedance/admittance monitoring system (WiAMS) that employs the voltage measurements of PZT transducers. Small-sized PZT patches that have been epoxy-bonded on the steel bars surface and on the external concrete face of the beams are utilized to diagnose damages caused by steel yielding and concrete cracking. Excitations and simultaneous measurements of the voltage signal responses of the PZT transducers have been carried out at different levels of the applied load during the tests using the developed SHM devices, which are remotely controlled by a terminal emulator. Each PZT output voltage versus frequency response is transferred wireless and in real-time. Statistical index values are calculated based on the signals of the PZT transducers to represent the differences between their baseline response at the healthy state of the beam and their response at each loading/damage level. Finite Element Modeling (FEM) simulation of the tested beams has also been performed to acquire numerical results concerning the internal cracks, the steel strains and the energy dissipation and instability parameters. FEM analyses are used to verify the experimental results and to support the visual observations for a more precise damage evaluation. Findings of this study indicate that the proposed SHM system with the implementation of two different PZT transducer settings can be effectively utilized for the assessment of structural damage caused by concrete cracking and steel yielding in flexural beams under monotonic and cyclic loading.

## 1. Introduction

An extreme event such as an earthquake can inflict extensive cracking and severe damage on reinforced concrete (RC) infrastructures. Besides, the structural resilience of RC members is also reduced over time due to gradual deterioration processes, such as ageing, reinforcement corrosion, fatigue and damage accumulation. In particular seismically-induced cracks adversely impact the capacity of RC structural members to withstand future earthquakes. The occurrence of wide cracking also leads to a decrease in the stiffness and durability of the member. Prompt and real-time diagnosis of cracking and evaluation of damage degree in existing RC structures would issue early warnings in order to prevent further deterioration decreasing the risk to catastrophic failures [1,2,3,4].

Within the field of structural health monitoring (SHM) of RC structures, several studies have been devoted to the topic of damage assessment and localization. Early non-destructive inspection techniques were based on the static displacement or strain response of structures or on the response of low-frequency vibrations of the structure by employing algorithms that include damage index methods or the change in stiffness and in flexibility [5,6,7,8]. Most of the proposed global static and dynamic response-based techniques proved either numerically complex, or difficult to apply to existing large-scale structures or not capable of detecting thin and local cracking [9]. Additionally, numerous local non-destructive techniques such as acoustic emission, ultrasonic techniques, eddy currents, impact echo testing, magnetic field, penetrant dye testing, infrared thermography and X-ray methods have been developed to diagnose or/and to localize cracks, irregularities and structural defects [10,11,12,13,14]. Each of these techniques has specific advantages and limitations, such as a need for prior knowledge or at least an approximation of the damage location, time-consuming inspection procedures and, therefore, difficulties to be applied to real-life structures [15,16,17].

Recent and advanced SHM techniques using smart materials and systems seem to be capable of providing continuous surveillance for the purposes of damage detection in concrete [18,19,20]. Piezoelectric lead zirconate titanate (PZT) patches have been widely implemented in recently developed SHM tedchniques due to their advantageous characteristics, such as small size, low cost, dual-functional capacity as both actuators and sensors, wide bandwidth and quick response. Electro-mechanical admittance (EMA) and its inverse electro-mechanical impedance (EMI) methods utilize these favorable features of PZT transducers and their mechanical interaction with the host infrastructure to provide feasible and practical applications in the SHM context [21,22,23,24,25,26].

Many scholars have conducted extensive research on the development and application of EMA-based SHM techniques combined with the implementation of PZT transducers for strength development in concrete [27,28,29,30,31,32,33,34], for corrosion in RC members [35,36], for damage detection in plain concrete [37,38,39,40,41,42,43,44,45,46,47,48,49], RC under monotonic loading [50,51,52,53,54,55,56], seismically valuable RC members under dynamic loading or lateral imposed cyclic deformations [57,58,59,60,61,62,63], prestressed concrete [64,65], steel [54,66,67,68,69,70,71], timber [72], steel-concrete composite [62,63,73] and composite [74,75,76,77] structural members.

Recent developments in SHM techniques have increased the efficiency of structural integrity assessment procedures. Most of the developed EMA-based SHM methods are rather complex, costly, non-portable, non-easy-to-apply in existing large-scale civil engineering structures and, therefore, not widely used in practical engineering. However, there are some, rather a few, available portable and wireless impedance-based monitoring systems in the literature. Min et al. [78] developed a low-cost, small-sized and wireless impedance sensor node based on the TinyOS platform as an operating system and controlled remotely via a MATLAB interface. It has been applied and checked to detect loose bolts and cracks in lab-scale and real-scale steel members of steel bridges and buildings. Park et al. [79] also used this sensor with connected PZT patches to diagnose the debonding conditions of carbon fiber reinforced polymer (C-FRP) plates that have been surface bonded in small-sized plain concrete prisms with promising results. Providakis et al. [80,81] developed an EMA-based wireless monitoring system that implements PZT transducers acting simultaneously as sensors and actuators to monitor early-age concrete strength gain and damages in plain concrete cylinders and prisms. Tzoura et al. [82] implemented the same wireless system to detect the debonding failure in 780 mm height RC columns retrofitted with C-FRP jackets. Hou et al. [83] imposed small-sized smart aggregates consisting of a single PZT transducer inside two marble blocks, under dynamic loading and measured its output signal using a wireless system. Perera et al. [84] developed an EMA-based wireless smart sensor framework using active PZT sensors for full-scale applications that has been tested on a lab-scale bolt jointed aluminum specimen and in a large-scale two-story two-bay steel frame with promising results.

Interesting research has also been carried out concerning the implementation of portable PZT-based active sensing boards in prestressed concrete. Nguyen and Kim [85] introduced a smart PZT-interface stress wave-based system using a multiplexed impedance sensor board to monitor prestress-loss in tendon-anchorage connection and Jiang et al. [86,87,88] proposed a data acquisition board connected with PZT patches and smart aggregates to detect the grouting quality of the prestressed curved tendon ducts in lab-scale test specimens. Zhang et al. [89] also implemented this data acquisition board to monitor the wedge anchorage system’s looseness status in steel strands with wedges and barrel anchorages.

In the aspect of PZT-based SHM techniques for crack-induced damage detection of large-scale structural members, the early study of Soh et al. [9] demonstrated the ability of this method to quantify damages in a large-scale RC bridge beam and to identify the local region in a bonded self-sensing PZT patch vicinity. Yang et al. [90] addressed the structural mechanical impedance extracted from the EMA signatures of PZT patches to indicate damage in a two-story RC frame under base vibrations. Kong et al. [91] tested real-scale RC bridge columns under simulated pseudo-dynamic lateral loading and developed a piezoelectric smart aggregate active sensing approach to detect crack propagation and in real-time. Sevillano et al. [92] combined the guided waves and EMI techniques as complementary to each other to also identify the debonding propagation of FRP-strengthened RC beam. In this direction, Sevillano et al. [93] and Perera et al. [17] extended their previous research and developed an interesting hierarchical clustering approach that collects and properly evaluates groups of complex measurements from a network of PZT transducers in order to identify damage in FRP strengthened RC structural members. Liao et al. [94] experimentally investigated the effectiveness of a multi-functional transducer device with post-embedded PZT sensors for local damage detection in real-scale RC frames under cyclic loading. Liu et al. [95] examined the debonding failure of RC beams strengthened with C-FRP plates.

Recent studies have highlighted that the installation of a network of multiple PZT transducers in areas nearby the potential damage of the examined structural member increases the effectiveness and the accuracy of the SHM method to identify damage providing a reliable diagnosis of its severity [17,96,97]. A newly developed wireless EMA-based monitoring system has been examined by the authors in shear-critical and slender RC beams under monotonic loading till failure and in one-story, one-bay RC frames subjected to lateral reversal cyclic deformations [98,99,100]. Various settings of PZT transducers such as embedded smart aggregates, internally bonded on the steel bars and bonded on the external concrete face have been implemented and their effectiveness and sensitivity in shear cracking damage detection have been checked. The results of these first experimental studies showed promising early indications of the forthcoming critical failures at early damage stages as the onset of diagonal cracking. However, questions have arisen concerning the ability of the developed SHM system to identify low-level damage that reaches more severely the internal areas of the structural member and to detect initial cracks before they become visible. Limitations on the quantification of damage assessment using statistical index values calculated from externally installed PZT sensors’ frequency signals have also been reported.

In the context of this research and in order to clarify the aforementioned issues and further examine the abilities/limitations of the developed innovative custom-made, portable, remote-controlled and low-cost EMA-based SHM system, the present study combines experimental and numerical investigations. The experimental part includes two large-scale RC flexural beams; one tested under monotonically increased loading and the other subjected to full cyclic reversal deformations. Two installations of internally and externally mounted PZT transducers are implemented to diagnose damages caused by steel yielding and concrete cracking. Finite element modeling (FEM) simulation of the tested beams is also included herein to acquire useful information concerning the internal cracks, the steel strains and the energy dissipation and instability parameters. Numerical results provide verification of the experimental results and support visual observations concerning the damage evaluation procedure with the examined SHM system.

This study also contributes to further establishing the implementation of EMA-based SHM systems in real-life existing large-scale RC structures for continuous inspection, early damage diagnosis and prompt warnings before fatal failures. The combination of FEM analysis and experiments addressing in this work provides to the research community an alternative SHM methodology. This approach includes an initial numerical investigation of the inspected, undamaged existing RC structure in order to acquire numerical damage indices in terms of internal energy and energy instability values. These numerical results determine preset values of damage parameters that would be used to alert the developed remote-controlled EMA-based SHM system in case of an extreme loading as seismic excitation that would cause severe cracking and structural damages in the monitoring RC structure.

## 2. Developed SHM System

**Figure 1:**
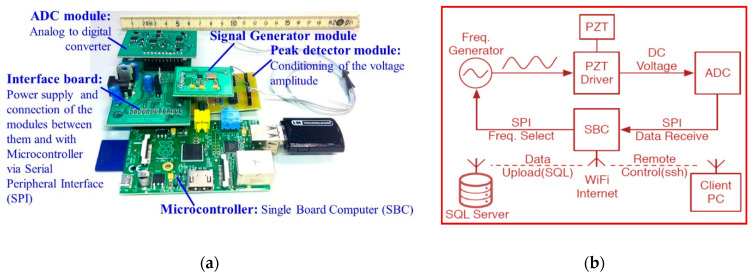
Instrumentation of the developed PZT-based SHM system (**a**) device; (**b**) block diagram of the interface board.

In this study, a real-time EMA sensing PZT-based SHM system is implemented for damage detection and evaluation. Called the wireless impedance/admittance monitoring system (WiAMS), it has been developed by Providakis et al. [40,41] and applied in RC structural members under monotonic loading by Voutetaki et al. [98] and under cyclic loading by Chalioris et al. [99,100]. Small-sized mobile custom-made devices compose the main hardware of the in-situ system that is shown in Figure 1. Each device is connected by two soldered wires with a small PZT patch that is mounted to the RC structural member in order to:

(1) vibrate the PZT transducer, that acts as an actuator, by an amplified harmonic excitation voltage,
(2) simultaneously monitor the signal of the PZT transducer, that receives the reflected waves acting as a sensor, in terms of electrical impedance,
(3) process the measured impedance values in terms of voltage frequency response, and
(4) transmit wireless and in real-time the final output response to the user using a Wi-Fi internet connection.

This integrated SHM system also enables full remote control via wireless fidelity (Wi-Fi) network connections, providing high processing power, email notifications, wireless data upload to structured query language (SQL) database, terminal emulator and iterative scheduled impedance measurements along with magnitude estimations. The device’s central control unit is a single board computer (SBC) Raspberry PI microcontroller that uses Linux as an operating system performing all necessary computing and interfacing tasks via serial peripheral interface (SPI). The developed WiAMS device comprises of two custom-made boards with the AD7357 analog-to-digital converter (ADC) and the AD9837 frequency generator, a custom-made power supply interface board that also connects the individual modules between them and with the central control unit and a piezo driver module. Further details and the numerical aspects that have been considered in the developed SHM system can be found in [40,41,98,99,100].

## 3. Experimental Program

### 3.1. Characteristics of the Beam Specimens and Installation of the PZT Transducers

**Figure 2:**
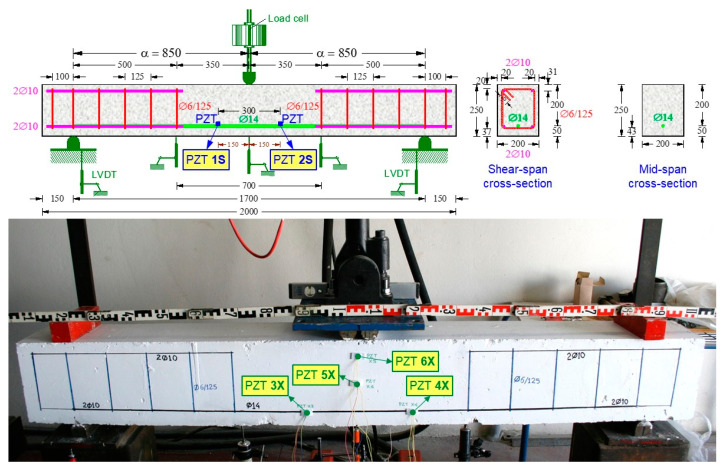
Characteristics of the tested RC FLM beam under monotonic loading.

**Figure 3:**
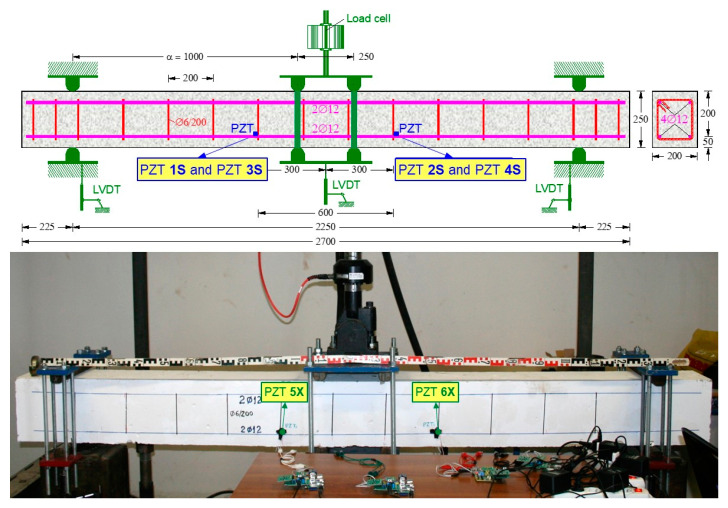
Characteristics of the tested RC FLC beam under cyclic loading.

The experimental program included two flexural large-scale RC beams. The flexure—monotonic (FLM) beam was subjected to monotonic loading, and the flexure—cyclic (FLC) beam was subjected to cyclic loading, as displayed in Figure 2 and Figure 3. The FLM beam had a total length *L_tot_* = 2000 mm, a net length *L_n_* = 1700 mm (support to support), while the ″FLC″ beam had *L_tot_* = 2700 mm and *L_n_* = 2250 mm, respectively. The cross-section of both beams was rectangular with width to height ratio *b*/*h* = 200 mm/250 mm, and the effective depth was *d* = 200 mm. The mean cylinder compressive strength of the concrete obtained from compression tests was *f_cm_* = 30.85 MPa and 28.20 MPa for the FLM and FLC beams, respectively.

The top longitudinal reinforcement of the FLM beam consisted of two (2) steel bars with a diameter of 10 mm (2Ø10) that extended only in the shear spans of the beam. The bottom longitudinal reinforcement of the FLM beam consisted of two steel bars with a diameter of 10 mm (2Ø10) extending only into the shear spans of the beam and one (1) bar with a diameter of 14 mm (Ø14) extending along its entire length. The beam also had transverse reinforcement in the form of closed stirrups, with a diameter of 6 mm placed only in the shear spans at 125 mm spacing (Ø6/125). The top and bottom longitudinal reinforcement of the FLC beam consisted of two (2) steel bars with a diameter of 12 mm (2Ø12 top and 2Ø12 bottom). The beam also had transverse reinforcement in the form of closed stirrups, with a diameter of 6 mm at equal 200 mm spacing (Ø6/200). The steel yield strength was measured as *fy* = 595 MPa and 550 MPa, for the Ø14 and the Ø12 longitudinal reinforcing bars of the FLM and FLC beams, respectively. Figure 2 and Figure 3 depict the geometry, the arrangement of the reinforcements and the test rig of the FLM and FLC beams, respectively.

Damage diagnosis of the tested RC beams during the loading procedure is achieved using the PZT voltage frequency responses measured by the developed portable SHM devices. Internally and externally installed PZT transducers are used. Especially, six (6) small-sized (10 × 10 mm^2^) and 2 mm thick PZT patches with material mark designation PIC 255 (PI Ceramic GmbH, Lederhose, Germany) are used in every RC beam as follows:

(i) Six (6) PZT patches have been epoxy-bonded on the steel surface of the tension reinforcing bars before concrete casting and located inside the tested RC beams:
  - The FLM beam includes two (2) internal PZTs denoted as “1S” and “2S” at locations shown in Figure 2.
  - The FLC beam includes four (4) internal PZTs denoted as “1S”, “2S”, “3S” and “4S” at locations shown in Figure 3.
(ii) Six (6) PZT patches have been epoxy-bonded on the concrete surface of the beams and located outside the tested RC beams:
  - The FLM beam includes four (4) external PZTs denoted as “3X”, “4X”, “5X” and “6X” at locations shown in Figure 2.
  - The FLC beam includes two (2) external PZTs denoted as “5X” and “6X” at locations shown in Figure 3.

**Figure 4:**
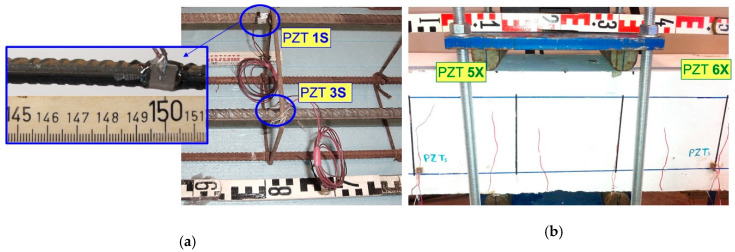
Installation of PZT patches (**a**) internally epoxy-bonded on the steel surface of the tension reinforcing bars; (**b**) externally epoxy-bonded to the concrete surface.

The epoxy used for bonding the PZT patches on the steel surface of the bars and on the concrete surface of the beams was a dual-component, high-strength, high-stiffness, fast-curing, water resistant and suitable for many materials and resin adhesive. A thin layer, approximately equal to one-third of the 2 mm thick PZT patch, of this epoxy adhesive has been applied. Findings of several types of research suggest that the adhesive could influence the capability of the bonded sensor to excite the structure and might affect the quality and repeatability of the EMI signals. Therefore, adhesive with a small thickness and high shear modulus is more appropriate to ignore its contribution to the interaction between the sensor and the host structure. Further, for long-term environmental exposure, epoxy adhesives are recommended in real RC structures [101,102,103,104]. Close up views of the installed epoxy-bonded PZT patches on the surface of the steel reinforcing bars, before concrete casting, and on the external concrete surface of the beams are shown in Figure 4.

### 3.2. Experimental Setup and Loading

**Figure 5:**
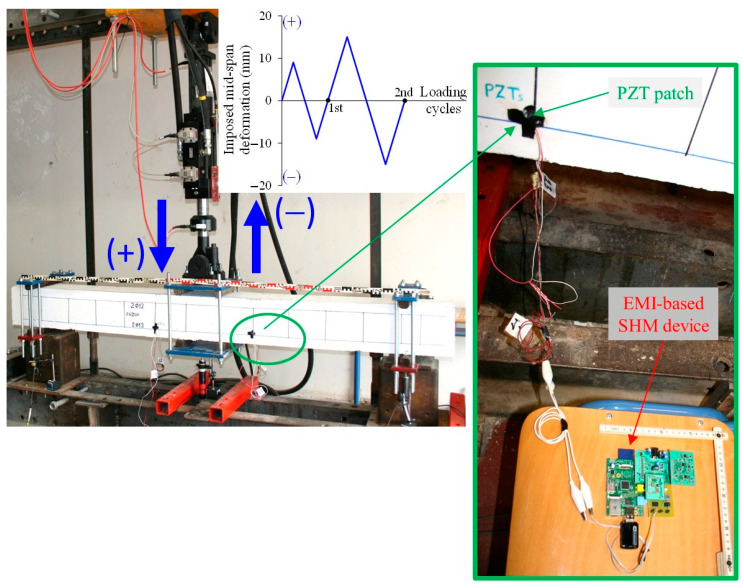
Test setup of the cyclic loading test, history and device of the developed SHM technique.

The FLM beam was subjected to a monotonic loading while the FLC beam was exposed to four-point cyclic bending loading. The overall experimental setup is shown in Figure 5. The beams were simply edge-supported on roller supports in a rigid laboratory frame. The loading was imposed by a rigid steel plate and was applied in the middle span of the beams. The loading was applied slowly and steadily, using a pinned end MTS actuator with a maximum load capacity of 100 kN and a maximum displacement of 250 mm (±125 mm). The loading was measured by a load cell attached to the actuator with ultimate load capacity 100 kN and accuracy 0.05 kN (MTS, Eden Prairie, MN, USA).

The displacements were measured using linear variable differential transducers (LVDTs) with a rated capacity of 50 mm and 100 mm, and 0.01 mm accuracy (Kyowa, Tokyo, Japan). Specifically, for the FLM beam, one LVDT with rated capacity 100 mm was placed in the middle of the bending span, two LVDTs with rated capacity 100 mm at distances of 500 mm from each support and one LVDT with rated capacity 50 mm at each support (see also Figure 2). For the FLC beam, one LVDT with rated capacity 100 mm was placed in the middle span and one LVDT with rated capacity 50 mm on each support (see also Figure 3). The imposed load and the corresponding displacements were controlled and recorded during the whole loading process using an integrated MTS FlexTest controller and data acquisition system.

The applied cyclic loading history is shown in Figure 5 and includes two mid-span deformation steps at ±75%*δ_y_* and ±125%*δ_y_*, where *δ_y_* is the deformation at yield. The installed PZT transducers have been excited by an amplified harmonic voltage of 10 V in the time domain range at every central frequency and their voltage signal responses were measured using the developed portable EMA-based SHM devices at different loading steps and at corresponding damage levels (see also Figure 5).

## 4. Finite Element Modeling (FEM)

FEM was conducted to track the damage evolution of the beams and correlate it with the damage tracked with the PZT and to verify their applicability. The commercial software ABAQUS 2017 [105] was used to simulate the specimens. Numerical analysis was conducted for both beams, and quasi-static loading was applied. The damage, as well as the hysteretic curves, were analyzed. Details of the simulation process are provided in the following section.

### 4.1. Material Properties

In the current study, the smeared cracked method was used to simulate the cracking of concrete. This approach is used because no predefinition of the crack paths is needed. In the smeared crack model, the geometry and therefore, the mesh remain unaltered, and the cracking of concrete is described through the constitutive relationships.

#### 4.1.1. Compressive and Tensile Behavior Constitutive Laws

**Figure 6:**
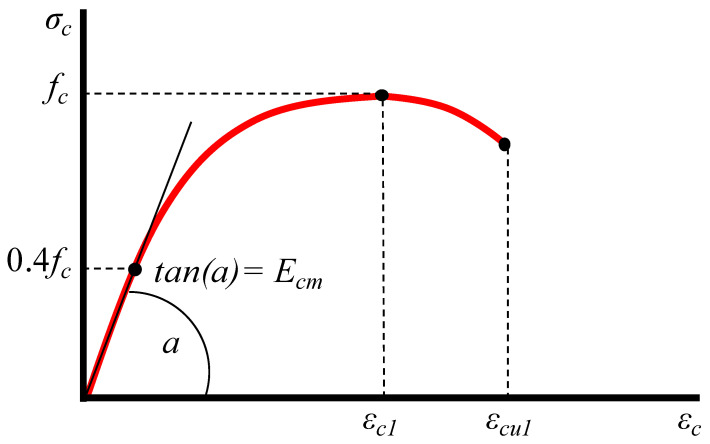
Stress-strain diagram of concrete under compression.

The compressive behavior of concrete is described by employing the stress-strain relationship for non-linear structural analysis provided by EC2 [106] and shown in Figure 6. The relation between *σ_c_* and *ε_c_* is described by the following expression:

(1) $$\frac{\sigma_{c}}{f_{cm}}=\frac{k\eta -\eta^{2}}{1+\left(k-2\right)\eta }$$

where *η* = *ε_c_*/*ε_c1_*, *ε_c1_* is the strain at peak stress and *k* = 1.05*E_cm_*|*ε_c1_*|/*f_cm_*. It is noted that this expression is valid for 0 < |*ε_c_*| < |*ε_cu1_*|, where *ε_cu1_* is the nominal ultimate strain.

For the tensile behavior of concrete, a smeared crack model, which had previously been established and validated experimentally by the authors [107,108] to predict the behavior of plain concrete under tension, has merely been modified considering the tensile stress-strain bilinear diagram proposed by Figueiras [109].

**Figure 7:**
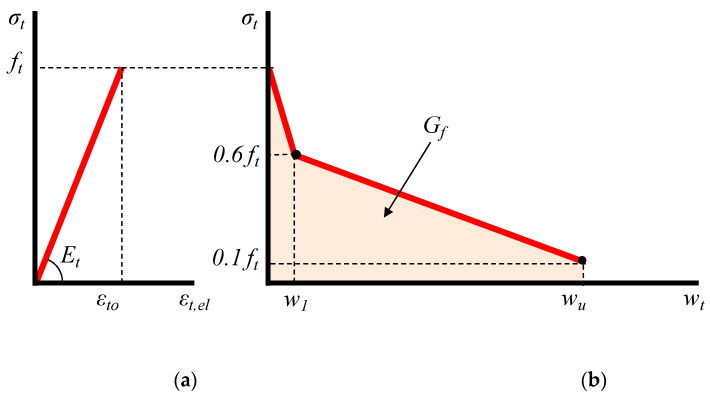
Tensile model of concrete including (**a**) stress versus strain elastic response and (**b**) stress versus crack width post-cracking response with tension softening.

The tensile behavior of concrete is defined by using constitutive laws in the context of normal stress versus crack width to describe the post-cracking response under tension instead of representing the cracking mechanism by stress-strain relationships. Such an analytical simulation has also been adopted in FEM to estimate crack width uncertainties in RC beams [110]. On this basis, crack propagation of concrete occurs with the development of a fracture process zone, which is launched at the maximum concrete tensile strength, *f_t_*, and is defined by a gradual decrease of strength throughout deformation. The boundary of the strain-softening area is characterized as this fracture process zone, which is a property attributed to RC and is assumed to be wider than the zone of visible cracks. It is also assumed that there are less damaged or even elastic parts between the cracks in this zone. Hence, the total tensile strain, *ε_t_*, is assumed as the sum of an elastic, *ε_t,el_*, and a fracture component, *ε_t,fr_*, which can be calculated on the basis of following relationships (Figure 7):

(2) $$\varepsilon_{t}=\varepsilon_{t,el}+\varepsilon_{t,fr},$$

(3) $$\varepsilon_{t,el}=\sigma_{t}/E_{t,},$$

(4) $$\varepsilon_{t,fr}=w_{t}/L_{fr}$$

where *σ_t_* is the tensile stress, *E_t,_* is the modulus of elasticity under tension, *w_t_* is the crack width and *L_fr_* is the length of fracture process zone of the concrete.

#### 4.1.2. Fracture Response

The characteristics of the fracture and the tension softening response of concrete determine the fracture component parameters of the smeared crack model. The fracture energy, *G_f,_* is the energy needed for the formation of the cracks included in the fracture process zone as well as for the complete opening of a single crack for a unit area crack plane, and can be expressed as:

(5) $$G_{f}=\int_{f_{t}}^{0}\sigma_{t}dw_{t}\overset{w_{t}=L_{fr}\varepsilon_{t,fr}}{\to }G_{f}=L_{fr}\int_{f_{t}}^{0}\sigma_{t}d\varepsilon_{t,fr}.$$

A by-linear descending branch shown in Figure 7, defines the post-peak fracture response (*σ_t_*–*w_t_* curve of Figure 7). The fracture energy could also be described in terms of the area under the curve of concrete tensile stress versus crack width:

(6) $$G_{f}=0.45f_{t}w_{1}-0.35f_{t}w_{u}\to ,$$

(7) $$w_{1}=\frac{G_{f}}{0.45f_{t}}-\frac{0.35w_{u}}{0.45},$$

The fracture energy of concrete, *G_f_*, can also be simply expressed using the following relationship as proposed by the author in [107]:

(8) $$G_{f}=0.5f_{t}w_{u},$$

(9) $$w_{u}=\varepsilon_{tu,fr}L_{fr}\overset{\varepsilon_{tu,fr}=a_{fr}\varepsilon_{to}}{\to }w_{u}=a_{fr}\varepsilon_{to}L_{fr}\overset{\left(23\right)}{\to }w_{u}=a_{fr}L_{fr}\frac{f_{t}}{E_{t}},$$

where: *f_t_* is the maximum tensile strength, and *w_u_* is the ultimate crack width. Also, *a_fr_* is a coefficient that depends on the shape of the stress versus crack width diagram as well the nature and the size of the concrete aggregates, and it ranges from 2 to 8 [107]. In the current study is considered as *a_fr_ = 3.3*. Further, *L_fr_* is the length of the fracture process zone of concrete that is taken as equal to 3*d_g_* [111].

#### 4.1.3. Damage Modeling and Stiffness Degradation

To further describe the nonlinearity of concrete, the evolution of damage was also considered. The concrete was modeled in the context of the known concrete damaged plasticity (CDP) model presented in ABAQUS. The non-linear behavior of concrete is related to the mechanisms of damage and plasticity. In the CDP model, the concrete’s constitutive behavior is defined by the introduction of scalar damage variables. Multiple mechanisms such as strain softening, gradual deterioration, volumetric expansion etc., can characterize the plasticity behavior. These contribute to a decrease in the strength and stiffness of the concrete. Damage is typically associated with the loss of stiffness.

After the completion of the elastic stage the entering in the stage of damage is described with stiffness degradation. Thus, the modulus of elasticity is reduced to:

(10) $$E=\left(1-d\right)E_{o},$$

where: *E_o_* is the initial elastic modulus; *d* is the plastic damage factor which varies from 0 ≤ *d* ≤ 1, with zero indicating the undamaged material, to one indicating a complete loss of strength.

Lubliner et al. [112] states that plastic degradation occurs only within the softening range and the stiffness is proportional to the material’s cohesion. Using the following equation, the plastic damage factor it is calculated as:

(11) $$\frac{E}{E_{o}}=1-d=\frac{c}{c_{max}}\to d=1-\frac{c}{c_{max}}$$

where *c* is cohesion in the yield criteria, which is proportional to stress; and *c_max_* is proportional to the strength of the concrete.

Damage is described in the concrete damage plasticity model for both uniaxial tension and compression during the softening procedure. Two damage variables, *d_t_* and *d_c_*, that correspond to tensile and compressive damage, define the degradation of elastic stiffness in the softening state.

(12) $$d_{c}=1-\sigma_{c}/\sigma_{cu},$$

where *d_c_* is the compressive damage variable and *σ_cu_* = *f_cm_* (mean concrete compressive strength).

(13) $$d_{t}=1-\sigma_{t}/\sigma_{to},$$

where *d_t_* is the tensile damage variable and *σ_to_* = *f_ctm_* (mean concrete tensile strength).

**Figure 8:**
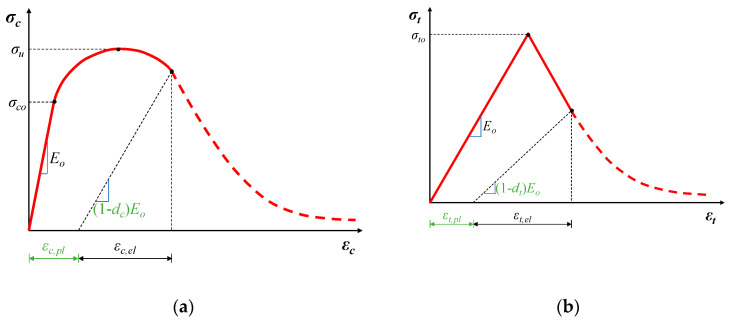
Definition of damage under (**a**) compression; (**b**) tension, where *ε_c,pl_* and *ε_t,pl_* are the equivalent plastic strains (considering damage) for compression and tension respectively and *ε_c,el_* and *ε_t,el_* are the elastic strains.

In the presented FEM, tensile and compressive damage introduced in the CDP model is considered to be in accordance with the previous equations and the diagrams in Figure 8. Full description of concrete behavior in the CDP model also requires the consideration of five plasticity parameters. The parameters definitions and the selected values in the current analysis are given in Table 1. More details on the CDP parameters can be found both in the ABAQUS user manual as well as in [113,114,115].

#### 4.1.4. Modeling of Steel Reinforcement

Elastic- perfectly plastic behavior was used to model the reinforcing steel material (longitudinal and stirrups). Before the yielding point the behavior was assumed elastic and was modeled using Young’s modulus according to test data values and Poisson’s ratio equivalent to 0.3. Plastic behavior after the excitation of yield point, was modeled using yield stress and corresponding plastic strain (*f_y_*, *ε_y_*) as well as the stress and strain at ultimate point (*f_u_*, *ε_u_*).

### 4.2. Simulation Characteristics

#### 4.2.1. Element Type

**Figure 9:**
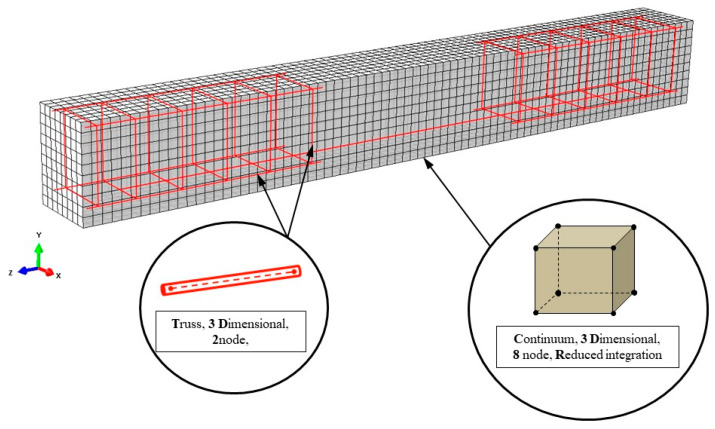
FEM mesh of concrete and steel reinforcement.

Both beams were simulated in three-dimensional space using the full geometry of the tested specimens shown in Figure 2 and Figure 3. In order to achieve accurate results with a rational computational time, all the elements, the concrete beam, steel bars and stirrups, were carefully simulated. Concrete was simulated using 3D eight node elements with reduced integration (C3D8R) and three degrees of freedom per node (see also Figure 9). Longitudinal reinforcement and stirrups were introduced as truss elements with three translational degrees of freedom (T3D2) at each node as shown in Figure 9. The steel reinforcement bending stiffness is assumed negligible compared to the RC matrix bending stiffness, thus is ignored. Therefore, the steel reinforcement is simulated using truss elements that can only carry axial forces. The interaction between concrete and reinforcement is modelled as perfect bond employing the embedded method.

#### 4.2.2. Mesh Size Selection—Convergence Study

**Figure 10:**
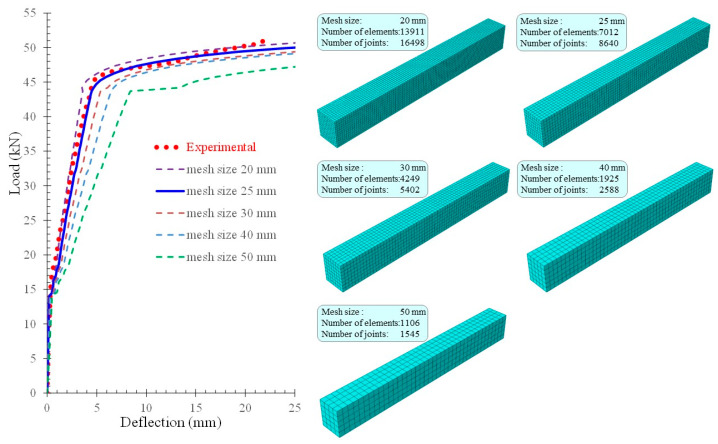
Parametric FEM analysis with different finite element sizes and corresponding mesh size details used for convergence study of the FLM beam.

In general, the coarser the finite element mesh grid is, the more accurate the solution is. This is because of the accuracy of the distribution of stresses and deformations in the structural members increases. However, a balance between the desired accuracy of the results and the analysis time should be achieved because, as the number of finite elements is increased (denser grid), the analysis time is longer. FEM analysis requires an investigation before selecting the final mesh size starting from a coarser grid and therefore larger finite elements and gradually decreasing the element size and moving to a finer grid. The solution’s convergence with the experimental results is studied until an appropriate size of finite elements is achieved. In the present study, concrete is the most sensitive material to the size of the finite elements due to its high nonlinearity and its overall response with the appearance of cracking and reduced stiffness. Figure 10 shows a comparison between the experimental result and the FEM simulation results obtained from the use of different sizes of finite elements for the beam ″FLM″. The parametric analysis showed that the use of finite elements of 30 mm size and smaller satisfactorily approaches the experimental behavior. The mesh size of 25 mm seems to provide the most accurate simulation of the experimental behavior, and thus it was finally selected for the analysis of the FLM beam. The convergence study was also carried out for the FLC beam, and in that case, the mesh size of 30 mm was the most suitable. Uniform mesh size for concrete and reinforcement elements was selected to make sure that different materials (steel and concrete) share common nodes.

#### 4.2.3. Load and Boundary Conditions Application

**Figure 11:**
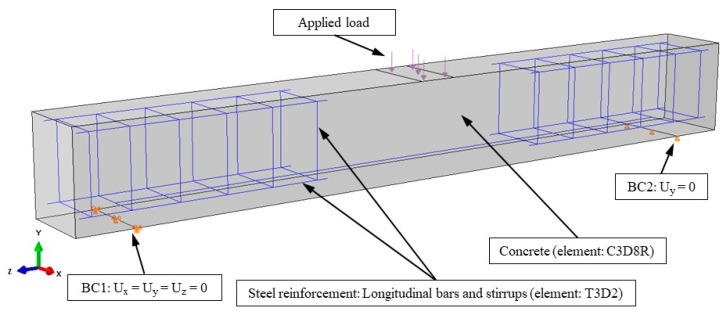
FEM boundary conditions, element types and applied load.

The beams were subjected to loading on their top face, which was simulated according to the experiment’s actual loading setup. The FLM beam was tested under monotonic bending loading, as shown in Figure 11, and the FLC beam under four-point cyclic bending loading. The load was applied steadily and gradually following the loading sequence of the experimental testing.

The boundary conditions were simulated equivalently to the supports of the experimental beam to ensure convergence. The left support was pinned (restricted in U_x_, U_y_, U_z_ directions), while the right support acted as a roller support (restricted only in U_y_ direction). This allows the beams to rotate freely at the supports.

#### 4.2.4. Material Input

The main material properties that are inserted in Abaqus to define concrete tensile behavior are presented in Table 2. Stress and crack widths and corresponding plastic damage factors shown in Table 2 are calculated using the equations presented above.

## 5. Results and Discussion

### 5.1. Experimental and FEM Simulation Results

**Figure 12:**
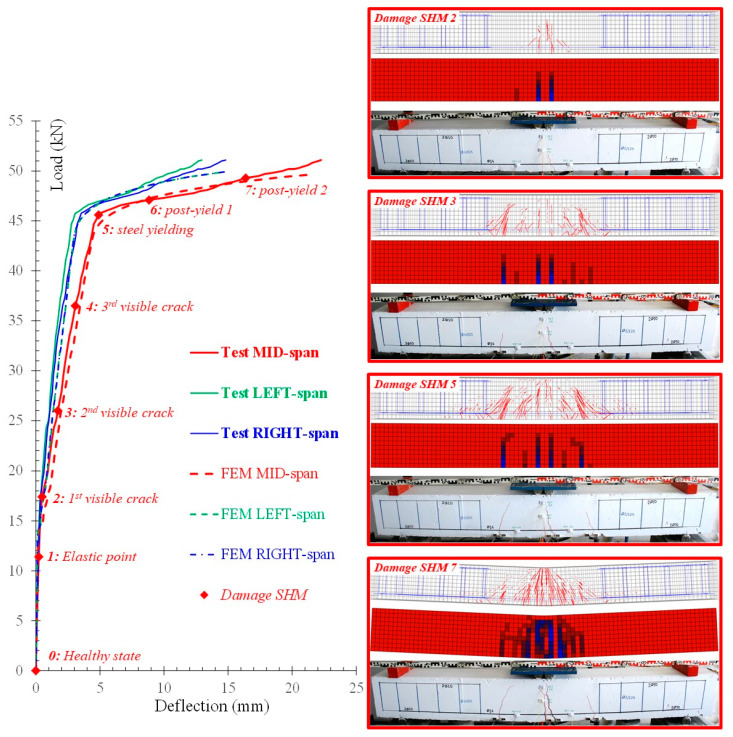
Comparisons between test and simulation results of the FLM beam under monotonic loading in load versus deflection curves and cracking patterns per various loading/damage levels.

The FLM beam tested under monotonic loading exhibited a flexural response as designed and expected. The experimental behavior of the beam is presented in Figure 12 in terms of load versus deflection curves in the mid-span, left-span and right-span. The applied load values and the corresponding net deflections of the beam have been evaluated using the measurements of the load cell and the LVDTs placed as described in Section 3.2. Specifically, the MID-span deflections have been calculated using the measurements of the LVDT placed in the middle of the bending span of the beam minus the corresponding mean measurements of the two LVDTs placed in each support. In the same manner, the LEFT-span and the RIGHT-span deflections have been calculated using the measurements of the LVDTs placed in the left and right span, 500 mm apart from each support, respectively, minus the corresponding mean measurements of the two LVDTs placed in each support. Measurements of the imposed load and the corresponding deflections were recorded simultaneously during the tests by an integrated data acquisition system.

For comparison reasons, the same diagram demonstrates the corresponding calculated load versus deflection curves derived from the FEM analysis. In Figure 12, typical cracking patterns of the experimental procedure are also presented and compared with the cracking patterns yielded from the performed numerical analysis using stress distribution data at the same loading level. The concrete tensile damage contour plots have also been provided to observe the failure modes of the beam. The red contour represents the undamaged concrete while the blue contour represents the concrete elements where d_t_ > 0. Crack propagation between test and FEM simulation at each loading step are in satisfactory compliance.

The flexural behavior of the FLM beam, shown in Figure 12, includes the following eight points of damage assessment using the proposed SHM technique: *0*: Healthy state of the unloaded beam, *1*: Elastic state, before cracking and at load 11.4 kN, *2*: First visible crack at load 17.4 kN (end of the elastic stage I), *3*: Second visible crack at load 26.0 kN, *4*: Third visible crack at load 36.5 kN, *5*: Steel yielding at load 45.7 kN (end of the pre-yield stage II), *6*: Post-yield (stage III) at load 47.1 kN and mid-span deformation 8.83 mm and *7*: Post-yield (stage III) at load 49.3 kN and mid-span deformation 16.37 mm. Each loading step corresponds to a different damage level that has been diagnosed using the proposed PZT-based SHM technique.

**Figure 13:**
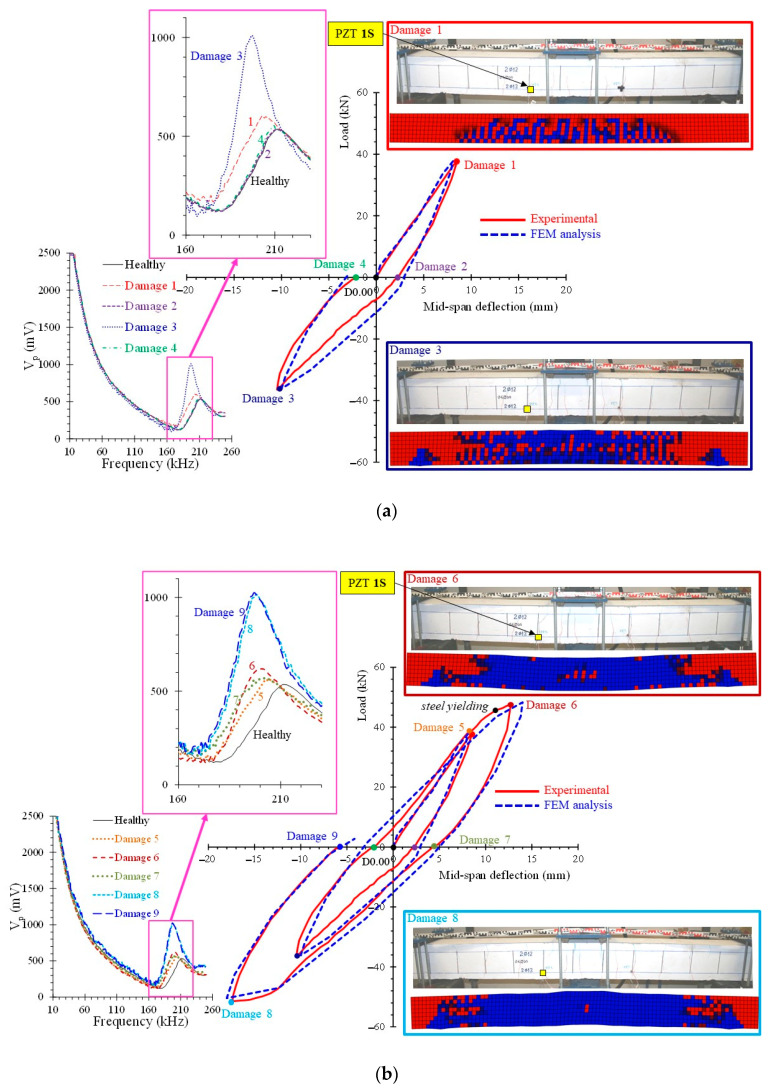
Comparisons between test and simulation results of the FLC beam under cyclic loading in load versus deflection curves and cracking patterns per various loading/damage levels for (**a**) the first loading cycle and voltage signal frequency response of the PZT 1S transducer; (**b**) both loading cycles and voltage signal frequency response of the PZT 1S transducer.

The experimental hysteretic response of the FLC beam subjected to cyclic loading is demonstrated in Figure 13. The first loading cycle is presented in Figure 13a in terms of load versus mid-span deflection curve and the hysteretic curves of both loading cycles are presented in Figure 13b. For comparison reasons, the same diagrams show the corresponding hysteretic curves calculated from the FEM simulation. Typical cracking patterns of the experimental cyclic loading are also presented in Figure 13 and they are compared with the numerical cracking patterns yielded from the performed FEM analysis using stress distribution data at the same loading level. The FEM contour plots refer to undamaged concrete elements (red color) and concrete elements where the tensile damage variable is d_t_ > 0 (blue color).

During the cyclic loading of the FLC beam the following twelve damage assessment procedures have been performed using the proposed SHM method (see also Figure 13 for notation): *0*: Healthy state of the unloaded beam, *Damage 1*: Maximum positive load and deformation of the first loading cycle, *Damage 2*: Unloading state of the positive first loading cycle, *Damage 3*: Maximum negative load and deformation of the first loading cycle, *Damage 4*: Unloading state of the negative first loading cycle, *Damage 5*: Positive load and deformation at 75% of yield deformation during the second loading cycle, *Damage 6*: Maximum positive load and deformation of the second loading cycle, *Damage 7*: Unloading state of the positive second loading cycle, *Damage 8*: Maximum negative load and deformation of the second loading cycle, *Damage 9*: Unloading state of the negative second loading cycle, *Damage 10*: Positive load and deformation at 75% of yield deformation during the final loading, *Damage 11*: Maximum positive load during the final loading till failure.

### 5.2. Damage Evaluation Results

Typical frequency responses of the performed SHM measurements of the PZT 1S transducer mounted on the steel reinforcing bar of the FLC beam in terms of voltage signal values per frequency range of excitation 10–260 kHz are presented and compared in Figure 13. Close-ups of these measurements are also presented in the same figure to discern the differences between the healthy and each damage level curves. Specifically, slight but certain differences can be observed in Figure 13a between the signal response for the “Healthy” state and the curves of “Damage 1” and “Damage 3” levels that correspond to the maximum positive and negative, respectively, load and deformation of the first loading cycle. On the contrary, the responses at the “Healthy” state and the “Damage 2” and “Damage 4” levels that correspond to the unloading state of the positive and negative, respectively, first loading cycle are more less the same. This is justified by the fact that PZT 1S is bonded on the steel tension reinforcing bar that remained elastic during the first loading cycle, thus no damage has occurred at the unloading state of “Damage 2” and “Damage 4” levels. However, obvious discrepancies of the signal responses of this transducer can be detected in Figure 13b between the baseline (healthy state) and most of the examined damage levels. Higher loading levels, such as “Damage 8” and “Damage 9” present the greater discrepancy.

The following known statistically damage index of the root mean square deviation (RMSD) is adopted to quantify damage assessment using the voltage signal measurements of the mounted PZT transducers:

(14) $$\mathrm{RMSD}=\sqrt{\frac{\sum_{1}^{N}{\left({\left|V_{p}\left(f\right)\right|}_{D}-{\left|V_{p}\left(f\right)\right|}_{0}\right)}^{2}}{\sum_{1}^{N}{\left({\left|V_{p}\left(f\right)\right|}_{0}\right)}^{2}}},$$

where *|V_p_(f)|*_0_ is the absolute value of the voltage signal measurements of the PZT at the healthy “baseline” state, *|V_p_(f)|*_D_ is the corresponding value at the examined damage level and N is the number of the measurements.

**Figure 14:**
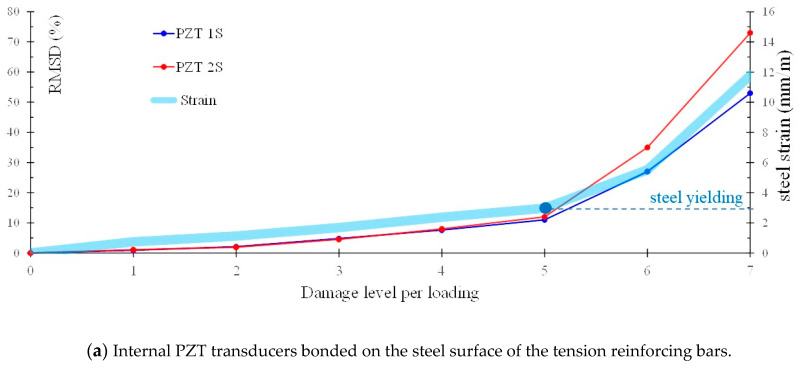

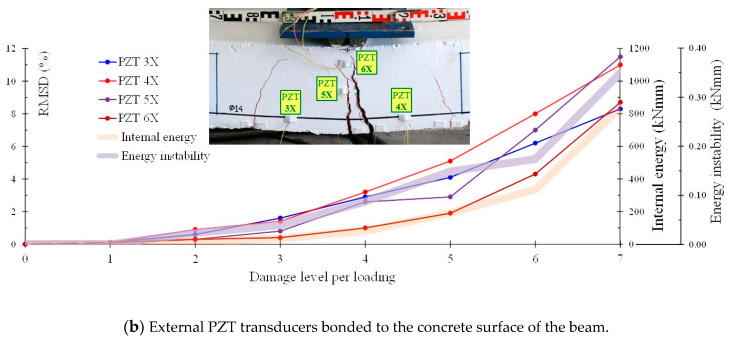
Values of RMSD index evaluated from the voltage frequency response signals of the PZT transducers for different level of loading/damage of the FLM beam.

**Figure 15:**
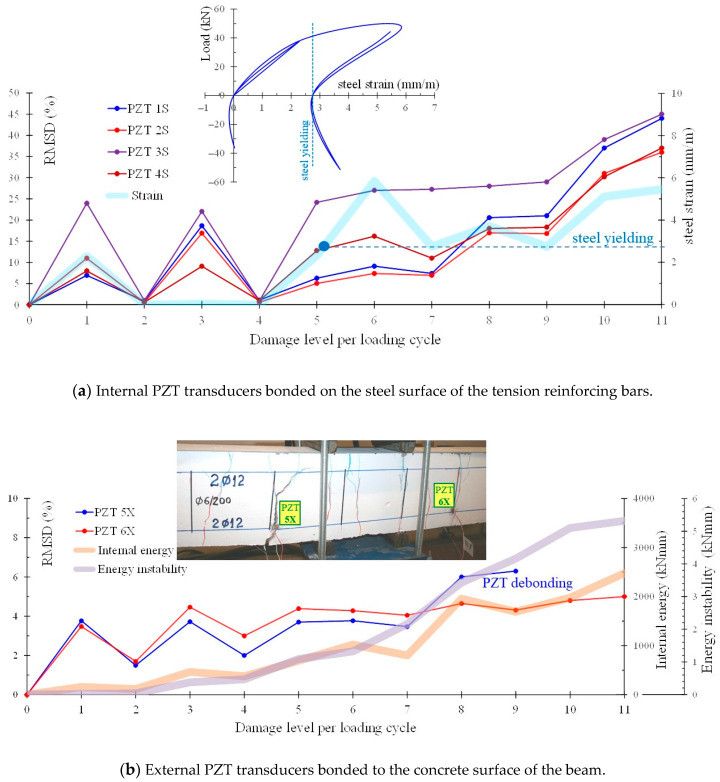
Values of RMSD index evaluated from the voltage frequency response signals of the PZT transducers for different level of loading/damage of the FLC beam.

Figure 14a,b show the variation of the RMSD index with the loading/damage level in the frequency range 10–260 kHz for the internally and externally installed PZT transducers, respectively, of the FLM beam. In the same manner, Figure 15a,b show the RMSD index values at each loading cycle for the internal PZT transducers bonded on the steel reinforcing bars and the external PZT transducers bonded to the concrete surface, respectively, of the FLC beam. In order to comprehend the results of the RMSD index in-depth, Figure 14a and Figure 15a also present the variation of the steel strain of the tension bars per loading step, and Figure 14b and Figure 15b present the values of internal energy and the energy instability in kNmm per each loading step. Steel strain, internal energy and energy instability values have been calculated by the performed FEM numerical analysis to establish the experimental results and to support the visual observations. Internal energy is the work applied by the external forces and energy instability is the dissipated energy released when a crack is extended by a certain amount. Each of the energy quantities has been directly requested and plotted at each time step from the FEM analysis software [105].

In Figure 14a it is noted that until loading step 5, viz. before steel yielding, the increase in RMSD index was approximately 10% and thereafter, there was a sudden and gradual increase in the value of RMSD index to about 30% at loading level 6 and to 60% at loading level 7 where tension steel bar of beam “FLM” yielded and the main flexural cracks were widened.

In Figure 14b, the gradual increase of the internal energy and energy instability during the monotonic loading procedure, that correspond to the increase of the energy dissipated by damage, seems to be followed by a similar increase of the RMSD index values derived from the signal responses of the externally surface-bonded PZT patches. Thus, cracks that occur during the flexural test cause a change to the voltage response of the external PZT transducers located to the region of the beam “FLM” near to damage/cracking. Crack propagation and the gradual opening of the cracks result in a progressive increase of the internal strain energy and, consequently to higher levels of damage, which is captured by the voltage signals of the PZT transducers that, accordingly, increase the values of the RMSD index. The cracking pattern at the failure of the FLM beam, which is displayed in an additional close-up photograph of the mid-span of the beam in Figure 14b, clearly indicates a typical flexural failure mode. It is stressed that visible cracks do not justify the high RMSD index values obtained by the voltage signal frequency response of the PZT 3X and PZT 4X” transducers. However, the calculated values of the internal energy and energy instability shown in Figure 14b along with the cracking patterns obtained from the FEM numerical analysis and displayed in Figure 12 confirm the variation of the RMSD index of these two PZT transducers. Thus, the combination of experimental and numerical results could lead to more safe conclusions concerning the effectiveness of the proposed SHM method.

In Figure 15a it is noticed that RMSD index values of the internal PZT transducers are more or less zero at the unloading steps of the first loading cycle of the FLC beam and before steel yielding, while increased RMSD index values can be observed in the second loading cycle and after steel yielding. Further, the RMSD index variation shown Figure 15b provides sound indications that the phenomenon of crack opening and closing during cyclic reversal loading can be captured effectively by the signal responses of the external PZT patches since their RMSD index values exhibit corresponding increases during crack opening (loading and reloading in “Damage 1” and “Damage 3” levels, respectively) and decreases during crack closing (unloading in “Damage 2” and “Damage 4” levels). Nevertheless, extensive cracking in larger deformation cycles could incapacitate or detach the PZT sensors located near the damage, such as PZT 5X (see also the diagram and the close-up photograph of the beam “FLC” at failure in Figure 15b).

The previous remarks provide sound indications that the proposed SHM technique with the implementation of two different PZT transducer settings can be effectively utilized to access of structural damage caused by concrete cracking and steel yielding in flexural beams under monotonic and cyclic loading.

## 6. Conclusions

The effectiveness of a custom-made, portable and low-cost EMA-based SHM system that utilizes PZT transducers in various settings to diagnose flexural damages caused by concrete cracking and steel yielding is demonstrated in this study. The experimental project includes two large-scale RC flexural beams subjected to monotonically increased loading until failure and to cyclic reversal deformations. Small-sized PZT patches are have been epoxy-bonded: (a) on the steel surface of the tension reinforcing bars as pre-installed internal transducers before concrete casting and (b) on the external concrete face of the beams. Excitations and simultaneous measurements of the voltage signal responses of the installed PZT transducers have been carried out at different levels of the applied load during the tests using the developed SHM devices, which are remotely controlled by a terminal emulator. Each PZT output voltage versus frequency response is transferred wireless and in real-time to the user of the monitoring system. Values of the known statistical index RMSD are calculated based on the signals of the PZT transducers to represent the differences between their baseline response at the healthy state of the beam and their response at each loading/damage level. Further, FEM simulations of the tested beams have been performed to acquire useful information concerning the internal cracks, the steel strains and the energy dissipation and instability parameters. Numerical results are used herein to verify the experimental ones and support visual observations concerning a more precise evaluation of damage with the examined SHM system.

In the case of the monotonically loaded beam, the variation of RMSD damage index acquired from the internal PZT patches bonded on the tension steel bars versus the loading level presents a sudden and gradual increase after the steel yielding point which is a clear indication of damage. Concerning the RMSD index values of the external PZT patches bonded on the concrete face of the beam, an increased tendency of these values according to the gradual opening of the flexural cracks during loading has been observed. However, propagation and magnitude of visible cracks seem not to justify the high RMSD index values obtained from some of the external transducers. In these cases, the numerical results derived from the FEM analysis provide variation of the dissipated energy and cracking patterns using stress distribution data that verify the RMSD index values, indicating that the damage has probably reached more severely the internal area. Thus, the combined experimental and numerical results lead to more safe conclusions about the effectiveness of the proposed SHM method.

In the case of the RC beam under cyclic loading deformations, the RMSD index variation of the internal PZT patches versus the loading steps seems to follow the variation of the tension steel bar strain up to the point of steel yielding. After this point, the frequency response of most PZT transducers exhibits high discrepancies with the baseline signal at a healthy state. Although extensive cracking in larger imposed deformation caused the detachment of the external PZT patch from the concrete surface, the crack opening and closing phenomenon during the cyclic reversal tests is captured effectively by the signal responses of these transducers. The RMSD index values derived from the external PZT patched present increases during crack opening due to loading and decreases during crack closing due to unloading of the beam.

The combined results derived from the performed tests and FEM simulations indicate the ability of the proposed SHM method to qualitatively identify damage caused by concrete cracking and steel yielding in flexural beams under monotonic and cyclic loading. Quantitative damage assessment is also attempted using statistically scalar damage index variations. For future works, specific values of this index should be determined for severe structural damage initiation to serve as warnings for imminent failures.

## Figures and Tables

**Table 1:** Parameters input of the CDP model.

Definition	Notation	Selected Value
Dilation angle	*ψ*	45°
Ratio of the tensile to the compressive meridian and determines the shape of the yield surface.	*K_c_*	2/3
Ratio of the strength in the biaxial state to the strength in the uniaxial state.	*σ_b0_/σ_c0_*	1.16
Flow potential eccentricity	*∈*	0.10
Viscosity parameter	*μ*	0.0001

**Table 2:** Input parameters of the examined RCbeams.

Beam Name	*Ε* (GPa)	*ν*	*G_f_* (N/mm)	*ft* (MPa)	*ε_t_*_0_ (‰)	*w_1_* (mm)	*w_u_* (mm)	*d_t1_*	*d_t2_*
FLM	30.85	0.2	0.03	2.42	0.078	0.00827	0.02481	0.4	0.9
FLC	30.03	0.2	0.026	2.23	0.074	0.00783	0.02348	0.4	0.9

## Data Availability

The data presented in this study are available on request from the corresponding author.
